# T-DNA Tagging-Based Gain-of-Function of OsHKT1;4 Reinforces Na Exclusion from Leaves and Stems but Triggers Na Toxicity in Roots of Rice Under Salt Stress

**DOI:** 10.3390/ijms19010235

**Published:** 2018-01-12

**Authors:** Yuuka Oda, Natsuko I. Kobayashi, Keitaro Tanoi, Jian Feng Ma, Yukiko Itou, Maki Katsuhara, Takashi Itou, Tomoaki Horie

**Affiliations:** 1 Division of Applied Biology, Faculty of Textile Science and Technology, Shinshu University, 3-15-1, Tokida, Ueda, Nagano 386-8567, Japan; yuuka-oda@kurilon.co.jp (Y.O.); 17fs704k@shinshu-u.ac.jp (Y.I.); takaito@shinshu-u.ac.jp (T.I.); 2 Graduate School of Agricultural and Life Sciences, The University of Tokyo, 1-1-1, Yayoi, Bunkyo-ku, Tokyo 113-8657, Japan; anikoba@g.ecc.u-tokyo.ac.jp (N.I.K.); uktanoi@g.ecc.u-tokyo.ac.jp (K.T.); 3 PRESTO, Japan Science and Technology Agency (JST), 4-1-8 Honcho, Kawaguchi, Saitama 332-0012, Japan; 4 Institute of Plant Science and Resources, Okayama University, Chuo 2-20-1, Kurashiki 710-0046, Japan; maj@rib.okayama-u.ac.jp (J.F.M.); kmaki@okayama-u.ac.jp (M.K.)

**Keywords:** salt stress, high affinity K^+^ transporter (HKT), rice, Na^+^ exclusion

## Abstract

The high affinity K^+^ transporter 1;4 (HKT1;4) in rice (*Oryza sativa*), which shows Na^+^ selective transport with little K^+^ transport activity, has been suggested to be involved in reducing Na in leaves and stems under salt stress. However, detailed physiological roles of OsHKT1;4 remain unknown. Here, we have characterized a transfer DNA (T-DNA) insertion mutant line of rice, which overexpresses *OsHKT1*;*4*, owing to enhancer elements in the T-DNA, to gain an insight into the impact of OsHKT1;4 on salt tolerance of rice. The homozygous mutant (the O/E line) accumulated significantly lower concentrations of Na in young leaves, stems, and seeds than the sibling WT line under salt stress. Interestingly, however, the mutation rendered the O/E plants more salt sensitive than WT plants. Together with the evaluation of biomass of rice lines, rhizosphere acidification assays using a pH indicator bromocresol purple and ^22^NaCl tracer experiments have led to an assumption that roots of O/E plants suffered heavier damages from Na which excessively accumulated in the root due to increased activity of Na^+^ uptake and Na^+^ exclusion in the vasculature. Implications toward the application of the HKT1-mediated Na^+^ exclusion system to the breeding of salt tolerant crop cultivars will be discussed.

## 1. Introduction

Soil salinization represents the excessive accumulation of water-soluble salts in the rhizosphere, and when the electrical conductivity of its saturation extract (ECe) is more than 4 dS m^−1^, the soil is considered as saline that can cause salt stress to glycophytic plants [1]. Soil salinity significantly reduces crop yield due to the imposition of salt stress. High salt environments reduce water uptake by roots because of a reduction in the water potential and disturb ion homeostasis for essential inorganic nutrients, such as K^+^ [2,3]. Furthermore, salt stress induces the overaccumulation of toxic elements, such as Na and Cl in shoots, which in turn, triggers toxicity that disrupts key metabolic processes [4,5]. To reduce the impact of salt stress on crop production, breeding crop cultivars that show higher salt tolerance is of great importance in addition to the attempts in developing agronomic and engineering solutions [6]. 

A Na^+^ selective plasma membrane transporter AtHKT1;1 has been demonstrated to be indispensable for being resistant to salt stress in Arabidopsis [7,8,9]. Subsequently, a major physiological role of AtHKT1;1 was found to limit the Na^+^ loading into the xylem for preventing Na overaccumulation in leaves [10,11,12]. A salt tolerance quantitative trait locus (QTL), which governs the trait of lower Na and higher K concentrations of rice shoots under salt stress in an analogous manner to the AtHKT1;1-dependent mechanism, was revealed to be constituted of the *OsHKT1*;*5* gene encoding a class I Na^+^ selective transporter [13]. Detailed physiological roles of OsHKT1;5 have recently been elucidated that the plasma membrane-localized Na^+^ transporter achieves lowering Na concentrations in leaf blades by mediating Na^+^ absorption into parenchyma cells at the root xylem and the phloem of basal nodes under salt stress [14]. Salt tolerance QTL analyses using wheat cultivars have also led to the findings that superior xylem Na^+^ unloading systems controlled by *Nax1* and *Nax2* QTL can prevent from over-accumulating Na in leaf blades of near isogenic lines of wheat cultivars suffering salt stress [15]. In the case of wheat, however, *TmHKT1*;*4*-*A2* was strongly suggested to be the causal gene for the *Nax1* QTL in addition to *TmHKT1*;*5*-*A*, being the causal gene for the *Nax2* QTL [16,17]. Recent reports with genetic and bioinformatic approaches further revealed that OsHKT1;1, a class I Na^+^ selective transporter in rice [18], also contributes to Na^+^ exclusion from leaves of salt-stressed rice plants [19,20,21], for which detailed mechanisms are yet to be elucidated. These past findings suggest that HKT1-mediated Na exclusion from leaves appears to be mediated by multiple HKT1 transporters in at least monocot plants. 

As for the *OsHKT1*;*4* gene in rice, it has been suggested that the gene product contributes to Na exclusion from leaf blades at leaf sheaths more efficiently in salt tolerant landraces than in a japonica rice cultivar [22]. More recently, OsHKT1;4 that exhibits a Na^+^ selective transport property in heterologous cells was found to play a significant role in excluding Na from stems and flag leaves of a japonica rice cultivar with the xylem Na^+^ unloading activity at least in a stem tissue, although the contribution of the transporter in the leaf blade Na exclusion during the vegetative growth stage seems to be minor [23]. The whole picture of the physiological role of OsHKT1;4 in the mechanism of salt tolerance of rice plants over the growth stages and rice varieties remains elusive. 

In this study, we have characterized a unique mutant line of rice, which overexpresses *OsHKT1*;*4* because of T-DNA activation tagging. Interestingly, the mutant line exhibited higher sensitivity to salt stress than its sibling WT plants, regardless of a higher capacity of the mutant for Na exclusion from leaves, stems, and seeds. Our studies provide possible mechanisms for the distribution of Na^+^ and K^+^ in rice tissues, influenced by *OsHKT1*;*4* overexpression. Presented results also provide hints for increasing salt stress tolerance of crops by the reinforcement of Na exclusion from leaves. 

## 2. Results

### 2.1. Isolation of a T-DNA Insertion Mutant of Rice Overexpressing OsHKT1;4

**Figure 1:**
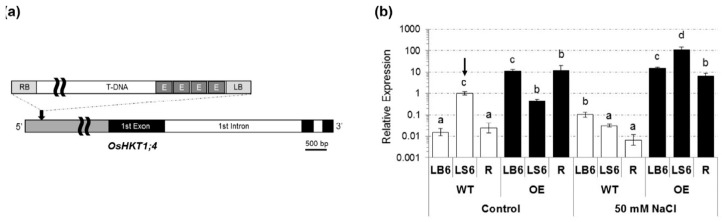
Isolation of a T-DNA insertion mutant rice line that overexpresses the *OsHKT1*;*4* gene, driven by enhancer elements in the T-DNA. (**a**) A schematic diagram of the T-DNA mutant allele used in this study, PFG_3A-05753.L. A grey box in the *OsHKT1*;*4* gene represents the promoter region, and exons and introns are indicated as black and white boxes, respectively. An arrow represents the insertion site of the T-DNA. Gray boxes with the word E in the T-DNA represent the enhancer element region, which is consisted of four tandem repeats of the core sequence of the 35S promoter. Note that the T-DNA insertion site is located at approximately 3 kb upstream from the first ATG of *OsHKT1*;*4*; (**b**) results of qPCR on the expression of *OsHKT1*;*4* in the 6th leaves and roots of the homozygous insertion line (O/E) and its sibling WT (WT). Approximately 3-week-old plants were prepared by hydroponic culture and 50 mM NaCl was imposed for 3 days prior to the experiment. The transcript level of *OsHKT1*;*4* and an internal control *OsSMT3* was analyzed. Relative expression of *OsHKT1*;*4* is shown with a logarithmic scale setting its expression in the 6th leaf sheath of WT from the control condition to 1 (*n* = 4, ±SD). LB6, LS6 and R represent, respectively, 6th leaf blades, 6th leaf sheaths, and roots. Different alphabets represent significant differences at the level of 5% (Tukey’s test).

We have searched the RiceGE database for potential *OsHKT1*;*4* mutants. We were interested in a line designated PFG_3A-05753.L as the T-DNA inserted upstream of the first ATG codon of *OsHKT1*;*4* harbors enhancer elements composed of tandem repeats of the 35S promoter core sequence [24] (Figure 1a). Homozygous insertion lines (O/E) and their sibling wildtype lines (WT) were isolated from the same parent line and the level of *OsHKT1*;*4* expression was investigated by the quantitative polymerase chain reaction (PCR) analysis (qPCR). The results indicated that O/E plants accumulated more than 100-folds of *OsHKT1*;*4* transcripts in the 6th leaf blades and roots compared with sibling WT plants even in the absence of salt stress (Figure 1b). Treatments of 50 mM NaCl on sibling WT plants triggered a trend of reduction in the accumulation of *OsHKT1*;*4* transcripts in the 6th leaf sheaths and roots (Figure 1b). In contrast, NaCl treatments on O/E plants significantly increased the level of *OsHKT1*;*4* transcripts in young leaf sheaths maintaining its level in young leaf blades and roots as high as those in the control condition (Figure 1b). 

### 2.2. OsHKT1;4 Overexpression Changes the Patterns of Na and K Accumulations in Tissues of Rice

**Figure 2:**
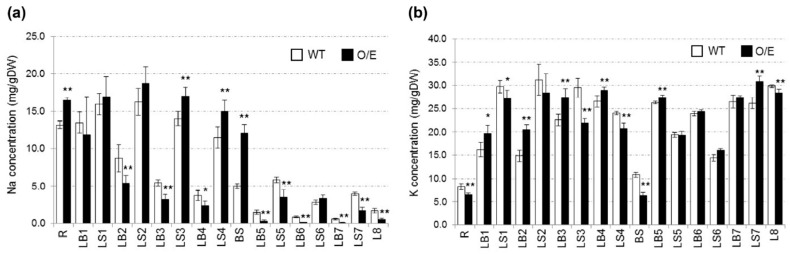
Measurements of Na and K concentrations using O/E and wildtype lines (WT) plants, which were subjected to salt stress. Plants were prepared by hydroponic culture, and 3 to 4-week-old plants were treated with 50 mM NaCl for 24 h. (**a**) Na concentrations in leaves, basal stems and roots; (**b**) K concentrations in the same tissues as in (**a**). LB, LS, BS and R represent, respectively, leaf blades, leaf sheaths, basal stems and roots. Larger numbers on LB and LS represent younger leaves. Asterisks indicate significant differences between O/E and WT plants (Student’s *t*-test: * *p* < 0.05, ** *p* < 0.01; *n* = 4, ±SD).

Concentrations of Na and K in tissues of approximately 3-week-old O/E and sibling WT plants, grown by hydroponic culture, were investigated. Treatments of 50 mM NaCl for 24 h led to higher concentrations of Na in sheaths of 2nd, 3rd, and 4th leaves of O/E plants than those of WT plants (Figure 2a). However, blades of those leaves of O/E plants showed significantly lower Na concentrations than WT (Figure 2a). Moreover, looking at younger leaves (5th to 8th), Na concentrations were significantly reduced in both sheaths and blades of the leaves of O/E plants in comparison with WT plants (Figure 2a). In roots and basal stems, O/E plants showed significant increases in the Na concentration (Figure 2a). 

As for K concentrations, relatively old leaves (1st to 4th), roots, and basal stems of O/E plants showed roughly an opposite trend to Na concentrations of the same tissues: that is, K concentrations were significantly increased in old blades, but decreased in old sheaths, roots, and basal stems of O/E plants in response to 50 mM NaCl (Figure 2b). In younger leaves (5th to 8th), K concentrations of O/E plants showed an increasing trend or remained unchanged when compared with WT plants (Figure 2b). 

**Figure 3:**
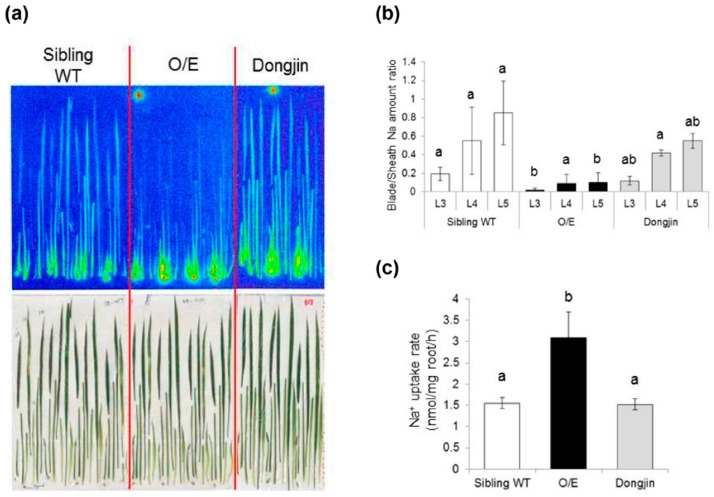
^22^Na tracer experiments using O/E and WT plants. (**a**) Allocation of ^22^Na in the shoot of rice plants. The autoradiograph (the upper image) and the picture of the sample (the lower image) were presented. All of the seedlings were composed of sheaths of the 1st leaf (L1), L2, and L3, L3 blade, L4 sheath, L4 blade, L5 sheath, L5 blade, L6, and the basal part of the shoot; (**b**) Na distribution ratios between the blade and the sheath of 3rd, 4th, and 5th leaves; (**c**) Sodium uptake rate (nmol/mg root/h) in the nutrient solution containing 10 mM NaCl. The means with standard deviations were presented (*n* = 3 for Dongjin and *n* = 4 for the O/E and the sibling WT). Different alphabets in (b) and (c) represent significant differences at the level of 5% (Tukey-Kramer method).

To further dissect the allocation of Na in O/E and WT plants, tracer experiments using ^22^NaCl were performed. Two-week-old plants, grown by hydroponic culture, were treated with 10 mM NaCl for three days prior to the experiment. ^22^Na radiation images of rice plants, obtained after 1 h absorption of ^22^NaCl from roots, indicated that O/E plants block ^22^Na^+^ transfer to leaves more efficiently holding more ^22^Na in the vicinity of basal nodes or at the bottom of sheaths than the sibling WT and Dongjin that is the background cultivar for this T-DNA mutant line (Figure 3a). Blade/sheath ^22^Na ratios of 3rd, 4th and 5th leaves showed a trend of lower ^22^Na allocation in leaf blades of O/E plants, supporting that O/E plants exhibit superior capacity for the blockage of ^22^Na^+^ transfer to leaves (Figure 3b). ^22^Na tracer analyses also led to the detection of significantly higher Na^+^ uptake rates (approximately a 2-fold increase) in the roots of O/E plants than others (Figure 3c). 

### 2.3. OsHKT1;4 O/E Rice Shows Increased Sensitivity to Salt Stress

**Figure 4:**
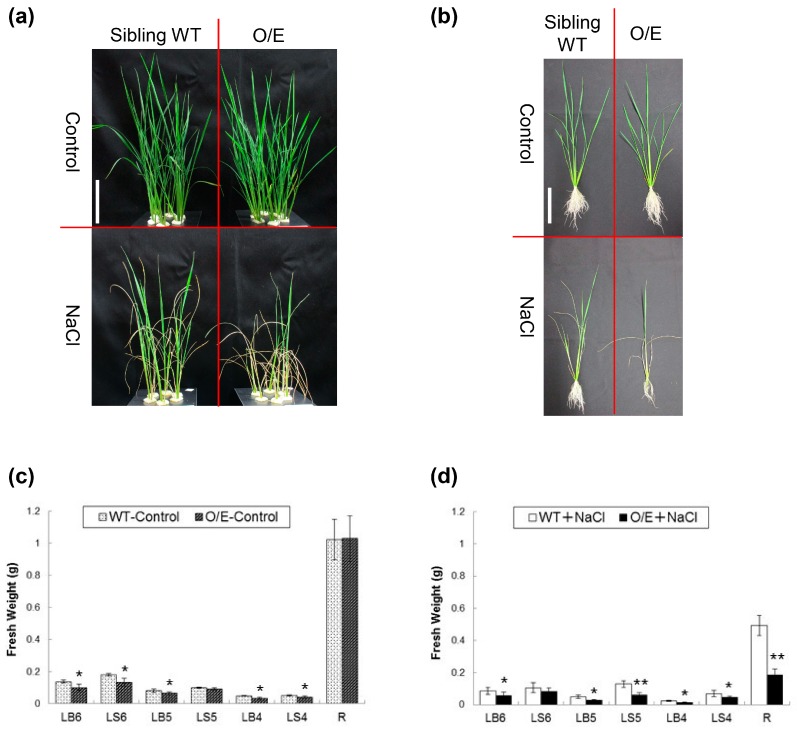
Increased salt sensitivity of the O/E line. (**a**) Pictures of O/E and sibling WT plants, which were prepared by hydroponic culture for approximately 2 weeks, and then treated with or without salt stress (a stepwise 25 mM increase in the NaCl concentration from 25 to 75 mM) for 9 days; (**b**) a representative plant from each condition mentioned in (**a**). Note that the white bars in (**a**) and (**b**) represent 10 cm; (**c**) fresh weights of leaf tissues and roots of O/E and WT plants, grown without salt stress (*n* = 6, ±SD); (**d**) fresh weights of leaf tissues and roots of O/E and WT plants, treated with salt stress (*n* = 6, ±SD). LB, LS, and R represent, respectively, leaf blades, leaf sheaths, and roots. Larger numbers on LB and LS represent younger leaves. Asterisks indicate significant differences between O/E and WT plants (Student’s *t*-test; * *p* < 0.05, ** *p* < 0.01).

Given that O/E plants exhibit more efficient Na exclusion from leaves under salt stress, which is known to be one of the positive factors for salt resistance, we compared the salt sensitivity of O/E plants with WT plants. Interestingly, salt stress obviously caused a heavier damage to O/E plants in visual than WT although both plants grew similarly without the stress (Figure 4a,b). Subsequent comparisons of the fresh weight of younger expanded-leaves and roots between O/E and WT plants revealed that NaCl treatments generally suppressed the growth of all the tissues tested, and, in particular, tissues from O/E plants suffered severer growth defects than WT (Figure 4c,d). Blades and sheaths of leaves of O/E plants resulted in showing approximately 21–54% reductions in the fresh weight when compared with WT tissues under salt stress (Figure 4d). However, the severest damage was found in roots of salt-stressed O/E plants with approximately a 62% reduction when compared to the roots of salt-stressed WT plants (Figure 4b,d) in contrast to the fact that roots of both lines did not show any difference under the control condition (Figure 4b,c). Note that 8–30% reductions in the fresh weight of leaves were also found in O/E plants when compared with WT tissues under the control condition, for which the reason is not yet clear, even though any visual symptom could not be observed during hydroponic culture without NaCl (Figure 4a–c). 

**Figure 5:**
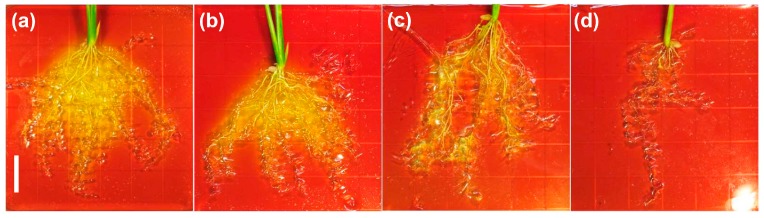
O/E plants show a decrease in the activity of H^+^-pump ATPases of the plasma membrane in response to salt stress. 10-day-old seedlings were treated with or without 50 mM NaCl for 7days. Then plants were transferred onto the 0.75% agar medium supplemented with 1 mM CaSO_4_, 2.5 mM K_2_SO_4_, and 0.006% bromocresol purple gently imbedding the roots on the surface of the medium. All agar plates were placed in a growth chamber under light for 6 h. Note that yellowing of the test plate represents acidification. Pictures were taken afterwards. 4–5 plants were used per condition and representative results were shown: (**a**) sibling WT plants without stress; (**b**) O/E plants without stress; (**c**) sibling WT plants with salt stress; and, (**d**) O/E plants with salt stress. Note that a white bar represents 1.5 cm.

To evaluate the biological activity of rice roots, we attempted to monitor the activity of plasma membrane H^+^-ATPases using bromocresol purple, a pH indicator. Approximately 10-day-old seedlings of O/E and sibling WT plants, as prepared by hydroponic culture, were treated with or without 50 mM NaCl for a week. Each plant was transferred to an agar plate including 0.006% bromocresol purple. As shown in Figure 5a,b, the color of the agar surrounding the roots of O/E and WT plants turned into yellow due to the acidification, which is an indication of active H^+^-pumping, and no noticeable difference was found between O/E and WT roots. However, when 50 mM NaCl-treated plants were transferred, the degree of the color-change was decreased in roots of both lines with more profound decreases in the case of O/E plants (Figure 5c,d). 

### 2.4. Influences of OsHKT1;4 Overexpression on Phenotypes of Rice Lines That Are in the Reproductive Growth Stage Under Salt Stress

**Figure 6:**
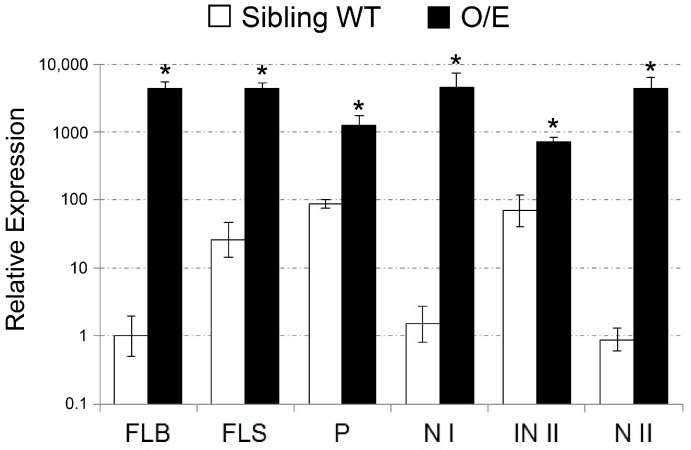
Results of qPCR on the expression of *OsHKT1*;*4* in tissues of the flag leaf and the stem of O/E and sibling WT plants, which were in the reproductive growth stage. Soil-grown plants were treated with salt stress by imposing a gradual 25 mM increase in the NaCl from 25 to 100 mM for more than a month. The transcript level of *OsHKT1*;*4* and an internal control *OsSMT3* was analyzed using the following tissues: FLB, the flag leaf blade; FLS, the flag leaf sheath; P, peduncle; N I, node I; IN II, internode II; and, N II, node II. Relative expression of *OsHKT1*;*4* is shown with a logarithmic scale setting its expression in the FLB of WT to 1 (*n* = 6, ±SD). An asterisk indicates significant differences between O/E and WT plants (Student’s *t*-test; * *p* < 0.001).

**Figure 7:**
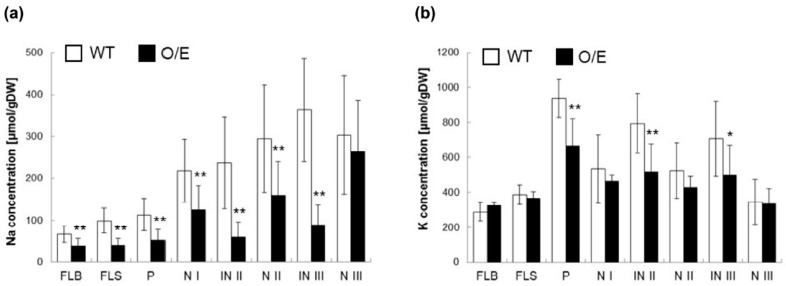
Measurements of Na and K concentrations using salt-stressed O/E and WT plants, which were in the reproductive growth stage. O/E and WT plants were grown in the same pot filled with the paddy filed soil, and, when the plants started heading, salt stress was imposed by gradually increasing the NaCl concentration in the tap water from 25 to 100 mM for more than a month. (**a**) Na concentrations in tissues of flag leaves and stems of O/E and WT plants (*n* = 12, ±SD); (**b**) K concentrations in the same tissues as in (**a**) (*n* = 12, ±SD). FLB, FLS, P, N I, IN II, N II, IN III, and N III represent, respectively, the flag leaf blade, the flag leaf sheath, peduncle, node I, internode II, node II, internode III, and node III. Asterisks indicate significant differences between O/E and WT plants (Student’s *t*-test; * *p* < 0.05, ** *p* < 0.01).

**Figure 8:**
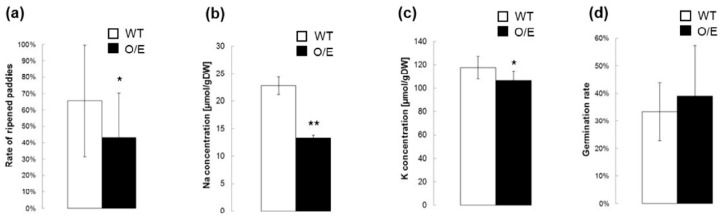
Effects of salt stress on the yield and the nature of the next generation seeds of O/E and WT plants. After the salt stress treatment with a gradual increase in the NaCl concentration of 25–100 mM for more than a month on soil-grown plants, they were watered with the tap water without the addition of NaCl. Resultant paddies were analyzed. (**a**) The rate of ripened paddies (*n* = 16 panicles for WT and *n* = 29 panicles for O/E, ±SD); (**b**) Na concentrations in ripened paddies (*n* = 9 experiments for WT and *n* = 18 experiments for O/E, ±SD); (**c**) K concentrations in ripened paddies (*n* = 9 experiments for WT and *n* = 18 experiments for O/E, ±SD); and, (**d**) Germination rate of the seeds from ripened paddies (*n* = 450 for each line, ±SD). Asterisks indicate significant differences between O/E and WT plants (Student’s *t*-test; * *p* < 0.05, ** *p* < 0.01).

We have further characterized O/E plants that are in the reproductive growth stage under salt stress since OsHKT1;4-mediated Na^+^ transport has been proposed to have a larger impact on the salt tolerance mechanism of rice during the reproductive growth stage [23]. We have at first analyzed whether O/E plants in the reproductive growth stage also show the feature of *OsHKT1*;*4* overexpression under salt stress. Both O/E and WT plants were grown in the same pot for approximately three months, and salt stress was imposed with a manner of a gradual increase in the NaCl concentration from 25 to 100 mM right after the initiation of heading for more than a month. qPCR using tissues of stems and flag leaves revealed substantial increases in the level of *OsHKT1*;*4* transcripts in tissues derived from O/E plants (Figure 6). We then measured Na and K concentrations in the tissues of stems and flag leaves of both lines, which were treated with salt stress as in the qPCR analysis. Except for the node III, significant decreases in the concentration of Na were found in tissues of O/E plants in comparison with WT (Figure 7a). As for the K concentration, peduncles, internode II, and internode III were found to show significant decreases (Figure 7b). The effect of salt stress on the rate of ripened paddies and their qualities was further evaluated imposing salt stress followed by a tap water irrigation for another month after the stress treatment. O/E plants turned out to show a significantly lower ripening rate than WT in response to salt stress, suggesting a severer damage of O/E plants than WT in the reproductive growth stage as well as in the vegetative growth stage (Figure 8a). Measurements of Na and K concentrations in ripened paddies indicated that both of the elements were significantly decreased in paddies from O/E plants when compared with those from WT (Figure 8b,c). However, no difference was found in the germination rate of the seeds from ripened paddies between O/E and WT plants (Figure 8d). 

## 3. Discussion

### 3.1. Influences of OsHKT1;4 Overexpression on the Salt Sensitivity and the Distribution of Na^+^ and K^+^ of the Rice Mutant Under Salt Stress

In this study, we have identified a T-DNA insertion mutant of rice that overexpresses *OsHKT1*;*4* (the O/E line) due to the function of enhancer elements in the T-DNA region [24], even though the insertion site is approximately 3kb upstream of the initiation codon of *OsHKT1*;*4* (Figure 1 and Figure 7). However, patterns of the accumulation of *OsHKT1*;*4* transcripts in young leaves of O/E plants in the vegetative growth stage were distinct, such that the accumulation level of the transcript in sheath was even decreased when compared with that of sibling WT plants under the control condition, while the level was significantly increased in O/E plants upon salt stress in opposition to the significant reductions of the level in WT plants by salinity (Figure 1b). Interestingly, the level of *OsHKT1*;*4* transcripts in leaf blades was kept significantly higher in O/E plants than in WT plants, irrespective of the NaCl concentration in the culture solution (Figure 1b). It is hard to explain the detailed mechanism that gave rise to these accumulation patterns in leaves of O/E plants, but one could consider a possibility that an overexpression process driven by enhancer elements in the T-DNA might have competed with the innate system driving the *OsHKT1*;*4* transcription in the leaf sheath, where the strongest expression of *OsHKT1*;*4* can be observed (Figure 1). As the transcript level in the sheath is significantly decreased in response to salt stress according to the qPCR result using WT plants, O/E plants might have had a chance to profoundly increase the transcript level in the sheath tissue by the effect of enhancer elements under salt stress (Figure 1b).

Measurements of Na and K concentrations and tracer experiments using ^22^NaCl demonstrated that O/E plants that are in the vegetative growth stage have an ability to exclude Na from young leaves more efficiently than WT plants under salt stress (Figure 2a and Figure 3a,b). The biggest factor that brought about such a phenotype of O/E plants appeared to be the more efficient blockage of Na^+^ transfer from roots to leaves (Figure 2a and Figure 3a), which was presumably achieved by the enhancement of OsHKT1;4-dependent Na^+^ exclusion in vasculatures of roots and basal nodes, including phloem parenchyma cells of diffuse vascular bundles in the basal node in addition to xylem parenchyma cells as has been demonstrated for OsHKT1;5 [14]. The feature of more efficient Na exclusion from leaves of plants is in general considered to be beneficial for being tolerant to salt stress [5,25]. Interestingly, however, O/E plants distinctly showed higher salt sensitivity than WT plants (Figure 4). Similar trends were also observed between O/E and WT when they were in the reproductive growth stage. O/E plants accumulated significantly lower concentrations of Na in stems and flag leaves than WT (Figure 7a), nevertheless soil grown O/E plants that were experienced with relatively a long-term salt stress turned out to exhibit significantly lower rates of ripened paddies compared to WT plants, which can be considered that O/E plants suffered a severer damage by salt stress, although the germination rate of the seeds from ripened paddies of both lines was similar (Figure 7a and Figure 8a,d). It is noteworthy that the constitutive overexpression of *AtHKT1*;*1* using the 35S promoter significantly increased the salt sensitivity of the Arabidopsis plants, however, unlike the case of the O/E line in this study, the transgenic plants accumulated more Na in leaves than WT [11]. 

Given that *OsHKT1*;*4* can be assumed to over-express in O/E plants over the growth stages (Figure 1 and Figure 7), it is intriguing that the enhancement of Na exclusion from leaves became a dominant phenotype in O/E plants under salt stress (Figure 2a and Figure 7a). Focusing on tissue Na concentrations of rice lines in the vegetative growth stage, Na concentrations were significantly decreased in leaf blades of old leaves (L2–L4), while those of leaf sheaths were increased (Figure 2a), suggesting enhanced Na^+^ exclusion around the xylem of the leaf sheaths. In contrast, in younger leaves (L5–L8), Na concentrations were significantly decreased in both blades and sheaths, implying that the toxic ions were less distributed to these leaves due to enhanced Na^+^ exclusion in vasculatures of roots and basal nodes (Figure 2a). Meanwhile, as for K, which is known to be an important factor determining plant salt tolerance [26,27,28], the pattern of the K accumulation in each tissue of old leaves (L1–L4) tended to be opposite to that of Na: that is, K concentrations significantly increases in blades, but decreases in sheaths (Figure 2b). In younger leaves, such an apparent tendency was weakened, however, some tissues show an increase in the K concentration in opposition to significant reductions in the Na concentration in the same tissues (Figure 2). These observations are nearly consistent with an assumption that Na^+^ unloading at the xylem often couples with those of K^+^ loading [25,29]. Focusing on rice lines in the reproductive growth stage, Na concentrations were significantly decreased in important aerial tissues of O/E plants when compared with WT plants, similar to the case of the vegetative growth stage (Figure 7a). The pattern of K concentrations, however, did not show an opposite trend to Na concentrations, and significant decreases in the K concentration were observed in peduncles, IN II and IN III (Figure 7b). We do not know the basis of these distribution patterns at this moment, but the mechanism of distributions of Na^+^ and K^+^ might become more complicated in the reproductive stage due to the development of a mature stem structure including nodes being a hub of complex mineral distributions [30].

### 3.2. Implications toward the Breeding of Salt Tolerant Crops by the Reinforcement of the HKT1-Mediated Na^+^ Exclusion System

The essentiality of the class I HKT-mediated Na^+^ unloading from the xylem for salt stress resistance has been firstly identified in Arabidopsis plants with the focus on the *AtHKT1*;*1* gene encoding a Na^+^ selective transporter [29]. Salt tolerance QTL analyses have revealed that class I transporters, both TmHKT1;4 and TmHKT1;5 in wheat and OsHKT1;5 in rice, function in a similar xylem Na^+^ loading mechanism to cope with salt stress in these crop plants [13,15,16,17]. OsHKT1;4 has been later revealed to contribute to Na exclusion from leaves and stems of rice suffering salt stress [22,23].

As discussed in the previous section, we presented evidence that *OsHKT1*;*4* overexpression increased the salt sensitivity despite the fact that the mutant line showed a superior leaf Na exclusion capacity to the sibling WT plants under salt stress (Figure 2a, Figure 3, Figure 4 and Figure 7a). We assume that these phenotypes found in O/E plants, which are apparently contradictory, might be caused by the overexpression of *OsHKT1*;*4* in all cells/tissues. O/E plants exhibited an approximately two-fold increase in the rate of Na^+^ uptake into roots (Figure 3c), suggesting that O/E plants would allow more rapid Na accumulation in roots than WT plants. When O/E plants were subjected to salt stress in the vegetative growth stage, the severest damage was observed in roots (Figure 4). Together with the indication of remarkably lower activity of H^+^-pump ATPases in the plasma membrane of roots upon salt stress (Figure 5), salt-induced significant increases in the Na concentration in roots of O/E plants (Figure 2a) suggest that a major cause for higher salt sensitivity of O/E plants can be attributed to a decrease in the biological activity of the root. It is therefore highly possible that higher rates of Na accumulation in roots of O/E plants, which was caused by the enhanced Na^+^ exclusion at the xylem and Na^+^ influx at surface tissues of roots, accelerated reaching to the threshold of the salt tolerance capacity of the root, over which Na toxicity appears. It is worthy to mention that salt-acclimation with a relatively low concentration of NaCl (25 mM) has been demonstrated to enhance salt tolerance of wheat plants [31]. In this study, we have imposed salt stress on rice lines starting with 25 mM or a lower concentration of NaCl on many occasions. Although it is not certain that salt-acclimation would have a positive influence on rice plants similar to the case of wheat, we assume that the way of phenotypic evaluation of O/E and WT plants in this study might avoid emphasizing on the effect of osmotic shock due to the sudden exposure to high salt concentrations, and that salt hypersensitive phenotypes of O/E plants, observed in this study, appear to be stable.

Taken all together, our study provides an insight that it is no use just reinforcing Na exclusion from leaves blindly to cope with salt stress, and that an allowable balance between Na exclusion from leaves and coordinated activity of vital roots must be maintained when the breeding of salt tolerant crops is considered using the enhancement of HKT1-dependent Na^+^ exclusion. On the other hand, if we manage to properly reinforce Na^+^ exclusion activity mediated by class I HKT transporters, including OsHKT1;4, such attempts could achieve increasing salt tolerance of crop cultivars with the feature of ideal leaf Na exclusion. In supporting the notion, introgression of the ancestral *TmHKT1*;*5*-*A* gene, the product of which mediates better Na exclusion from leaf blades, into a sensitive durum wheat cultivar has been proven to largely restore the yield of wheat grains in salt-affected agricultural lands [15,32,33]. Moreover, the enhancement of the *AtHKT1*;*1* expression in root stelar cells using the cell type specific enhancer trap system increased the salt tolerance of the transgenic Arabidopsis lines [11]. 

Note, however, that the physiological roles of OsHKT1;4 remain to be elucidated. Although OsHKT1;4 has been suggested to efficiently function in Na exclusion from young leaf blades of salt tolerant landraces in the vegetative growth stage [22], RNAi-mediated down-regulation of *OsHKT1*;*4* has later revealed that OsHKT1;4-mediated Na^+^ transport does not appear to have a significant impact on lowering the Na concentration of leaves of a japonica rice cultivar in the vegetative growth stage under salt stress, while in contrast, it does contribute to Na exclusion from stems and flag leaves of the same cultivar facing to high salt conditions when the growth stage was in the reproductive phase [23]. To gain better insights into the physiological role of OsHKT1;4 in rice and the potential use of the gene for the breeding of salt tolerant rice cultivars, elucidating precise tissue specificity of the OsHKT1;4 protein and the regulatory mechanism of *OsHKT1*;*4* expression in both salt tolerant landraces and sensitive cultivars will be essential.

## 4. Materials and Methods

### 4.1. Plant Material and Growth Conditions

The T-DNA insertion mutant of rice used in this study, PFG_3A-05753.L (*O. sativa* L. cv. Dongjin), was found at the Rice Functional Genomic Express Database (Available online: http://signal.salk.edu/cgi-bin/RiceGE). A homozygous mutant and its sibling WT lines were isolated from the same parent using specific primers (Table S1). Seeds were surface sterilized and germinated, as described previously [34]. For hydroponic culture of rice, seedlings were transferred to a plastic pot filled with the Kimura B nutrient solution and grown in the growth chamber, as described previously [23]. Indicated amounts of NaCl were added into the nutrient solution as salt stress and plants were further cultured for a certain amount of time depending on the experiment. On the other hand, seedlings were transferred to a plastic pot filled with the paddy filed soil and grown in a greenhouse to prepare rice plants that are in the reproductive growth stage. Salt stress was imposed for qPCR, measurements of Na and K concentrations, yield evaluation, and the test for the seed germination rate, as descried previously [14]. Note that, upon the sampling of the root, the whole root system was collected and then used for subsequent experiments. Furthermore, when the leaf blade and the leaf sheath were collected separately, special organs, such as a collar, which can easily be recognized in the boundary area between the blade and the sheath, were used as a marker to cut.

As for the determination of the rate of ripened paddies, three independent pots, each of which included one WT and two O/E plants, were prepared. All of the ripened paddies and non-ripened paddies were counted to calculate the rate, and the results were presented combining all the data sets. As for the determination of the germination rate, seeds from ripened paddies were germinated as mentioned above and kept for several days. The seeds that showed ordinal leaf development were considered as germinated ones and the rate was calculated. 

### 4.2. Total RNA Extraction and Real-Time PCR analysis

Total RNA was extracted from each tissue of O/E and WT plants using an RNeasy Plant Mini Kit (Qiagen, Limburg, The Netherlands). Reverse transcription reactions and subsequent real time PCR analyses were performed using PrimeScript™ RT Master Mix and a Thermal Cycler Dice Real Time System II TP800 (Takara, Japan), as described previously [23]. Primer sequences for *OsHKT1*;*4* and an internal control *OsSMT3* were shown in Table S1. 

### 4.3. Measurements of Na and K Concentrations in Tissues and Seeds of Rice

As for tissues from rice plants that were in the vegetative growth stage, samples were prepared, as described previously [35], and Na and K concentrations were measured by Inductively Coupled Plasma-Mass Spectrometry (ICP-MS) (7700X, Agilent Technologies, Santa Clara, CA, USA). As for tissues from rice plants that were in the reproductive growth stage, ICP samples were prepared, as described previously [23]. In the case of paddy samples, 10 paddies were selected at random from a panicle to make a batch of samples, and washed with ultrapure water. All of the paddy samples were dealt with as other tissues from rice in the reproductive stage [23]. Na and K concentrations were determined by Inductively Coupled Plasma-Optical Emission Spectrometry (ICP-OES) (Optima 7300DV, PerkinElmer, Waltham, MA, USA). 

### 4.4. ^22^Na Tracer Experiment

Two-week-old seedlings grown in the half-strength Kimura B nutrient solution were transferred to the nutrient solution containing 10 mM NaCl for three days. Then, the seedlings were placed in the nutrient solution supplied with 10 mM NaCl and ^22^Na (18.5 kBq/mL) for labeling. After labeling for 1 h, roots of the seedlings were dipped in the cold nutrient solution for 10 min to remove adsorbed ^22^Na. The labeled seedlings were cut into roots and shoots, and weighted. The amount of ^22^Na (cpm) in the root and in the labeled nutrient solution were measured using Gamma Counter (ARC-300, Aloka, Tokyo, Japan), and the amount of Na accumulated during 1 h was determined. The shoots were further separated into each leaf part and the ^22^Na distribution was quantitatively visualized using an imaging plate (BAS-IP-MS, GE Healthcare, Tokyo, Japan) and a FLA5000 image reader (FujiFilm, Tokyo, Japan). Sodium uptake rate was determined by dividing the total Na amount (nmol) accumulated in the whole plant during 1 h by the weight of the root (mg).

### 4.5. Detection of Rhizosphere Acidification Using a pH Indicator

Acidification of rhizosphere of rice plants was investigated referring to past reports [36,37], with some modifications. In brief, 10-day-old seedlings were prepared and transferred on a plastic pot containing a hydroponic culture solution supplemented with or without 50 mM NaCl. Seven days later, roots of the seedlings were washed with ultrapure water and transferred on the 0.75% (*w*/*v*) agar plate containing 1 mM CaSO_4_, 2.5 mM K_2_SO_4_, and 0.006% (*w*/*v*) bromocresol purple, pH 6. The roots of transferred plants were then gently imbedded into the agar. Several milliliters of a liquid solution containing 1 mM CaSO_4_, 2.5 mM K_2_SO_4_ were spread on the surface of the agar plate, and the plate was subsequently covered by a plastic wrap to avoid dehydration. They were incubated in a growth chamber under light for 6 h for visualization of rhizosphere acidification. 

## Supplementary Materials

Supplementary materials can be found at www.mdpi.com/1422-0067/19/1/235/s1.

## Abbreviations

HKT	high affinity K^+^ transporter
ICP-MS	inductively coupled plasma-mass spectrometry
ICP-OES	inductively coupled plasma-optical emission spectrometry
PCR	polymerase chain reaction
QTL	quantitative trait locus
RNAi	RNA interference
T-DNA	transfer DNA
