# Supplementary material for: T-DNA Tagging-Based Gain-of-Function of OsHKT1;4 Reinforces Na Exclusion from Leaves and Stems but Triggers Na Toxicity in Roots of Rice Under Salt Stress
Source: Int J Mol Sci. 2018 Jan 12;19(1):235. doi: 10.3390/ijms19010235 (PMC5796183; doi:10.3390/ijms19010235)
Supplement: Supplementary file 1 [file ijms-19-00235-s001.pdf]

Table S1. Primers used in this study.

Primer name	Seuqence (5' to 3')	Purpose
05753-F	TTGCAAACCTTTTAGGCACC	Isolation of homozygous insertion & sibling WT lines
05753-R	ACCACTGCTGCAGAAAACAC	Isolation of homozygous insertion & sibling WT lines
LB_T-DNA	ACGTCCGCAATGTGTTATTAA	Isolation of homozygous insertion & sibling WT lines
OsHKT1;4_Q-PCR-F	GTCGAAGTTGTCAGTGCATATGG	Amplification of *OsHKT1;4* transcripts in qPCR
OsHKT1;4_Q-PCR-R	TGAGCCTCCCAAAGAACATCAC	Amplification of *OsHKT1;4* transcripts in qPCR
OsSMT3-F	CCCACATCAACCTCAAGGTCA	Amplification of *OsSMT3* transcripts in qPCR (internal control)
OsSMT3-R	ACGGAGCCTACGACCATCAA	Amplification of *OsSMT3* transcripts in qPCR (internal control)
